# Randomized pilot trial of cell phone support to improve medication adherence among adolescents and young adults with chronic health conditions

**DOI:** 10.1186/s44247-024-00069-w

**Published:** 2024-03-19

**Authors:** Caitlin S. Sayegh, Karen K. MacDonell, Ellen Iverson, Breaon Beard, Nancy Chang, My H. Vu, Marvin Belzer

**Affiliations:** 1 Division of Adolescent and Young Adult Medicine, Children’s Hospital Los Angeles, 4650 Sunset Blvd., MS#2, Los Angeles, CA 90027, USA.; 2 Division of General Pediatrics, Children’s Hospital Los Angeles, Los Angeles, USA.; 3 Department of Pediatrics, University of Southern California Keck School of Medicine, Los Angeles, USA.; 4 Department of Behavioral Sciences and Social Medicine, Florida State University College of Medicine, Tallahassee, USA.; 5 Division of Hematology, Children’s Hospital Los Angeles, Los Angeles, USA.; 6 Division of Endocrinology, Children’s Hospital Los Angeles, Los Angeles, USA.; 7 Biostatistics Core, The Saban Research Institute, Children’s Hospital Los Angeles, Los Angeles, USA.

**Keywords:** Adherence, Mobile health, Cell phone, Text message, Coaching, Reminders, Chronic illness

## Abstract

**Introduction:**

Adolescents and young adults (AYA) living with chronic medical conditions often struggle to develop medication adherence skills. This pilot trial evaluated the impact of a mobile health coaching intervention, Cell Phone Support (CPS), on medication adherence.

**Methods:**

Interventions in this randomized trial were CPS delivered by phone calls (CPS-C), CPS delivered by text messages (CPS-T), or automated text message reminders (ATR). Participants were AYA with different chronic medical conditions (i.e., sickle cell disease, solid organ transplant, type 2 diabetes), aged 15–20 years (*N* = 34). We examined the feasibility, acceptability, and preliminary efficacy of each intervention.

**Results:**

We examined the feasibility, acceptability, and preliminary efficacy of both CPS interventions. CPS was feasible and acceptable. There was evidence that participants found CPS to be more useful than ATR. In this pilot trial, participants receiving CPS reported relatively stronger increases in adherence, compared to those assigned to ATR. CPS-C slightly outperformed CPS-T.

**Conclusions:**

Providing coaching to AYA struggling with illness self-management via their cell phones may promote their acquisition of medication adherence skills. Although larger studies are needed to confirm the results of this pilot study, phone calls and text messages are both promising modalities for delivering human cell phone support.

**Trial registration:**

This trial was registered prospectively at ClinicalTrials.gov (NCT04241627) on 1/27/2020.

## Background

In the US, there are over 10 million youth living with chronic health conditions (CHC), many of whom require daily oral medication [1]. Over half of adolescents and young adults (AYA) with CHC are nonadherent to their medication, resulting in detrimental health outcomes, increased healthcare expenditures, and poorer quality of life [2]. For AYA, achieving mastery of illness self-management skills, like adherence, is a dynamic developmental process with ramifications for their health and wellbeing in adulthood [3, 4].

Since over 95% of AYA use cell phones [5], mobile health (mHealth) technology is a promising method for delivering self-management interventions. To date, several mHealth interventions have demonstrated efficacy for improving medication adherence [6]. Of the existing adherence-promoting mHealth interventions, nearly all are disease-specific [6] which may limit patient access to effective support, particularly for those living with rare or less studied health conditions. A general, flexible mHealth intervention for AYA living with different CHC could improve the scalability of adherence-promoting interventions.

Cell Phone Support (CPS) is an adherence-promoting mHealth intervention that has been piloted separately with AYA with HIV [7] and organ transplants [8] showing signals of efficacy for increasing medication adherence. CPS includes short phone calls (< 5 min) made several times a week by a human coach to provide social support, medication reminders, problem-solving coaching, incentives for answering calls, and referrals to other services. Pilot results indicate that CPS may promote adherence in two very different medical conditions; however, national trends in technology use suggest phone calls may be less acceptable to today’s youth than other communication mediums. AYA report believing text messaging is easier, faster, less socially awkward, and more confidential than talking on the phone [9]. Although some promising two-way, interactive text message adherence interventions exist [10] most have been automated interventions, not live, real-time conversations between human coaches and AYA patients [11]. However, in a recent trial, youth living with HIV who received a multi-component adherence intervention which included two-way interactive out-reach texts from a human coach showed greater medication adherence compared to standard care [12].

Although AYA may prefer texting to phone calls, it is unknown how delivering CPS through this communication medium could impact its effects. What most distinguishes CPS from other mHealth interventions is that human coaches deliver the intervention in real-time, live phone calls. The Supportive Accountability Model provides a framework for understanding how technological aspects of mHealth could impact the provision of support [13]. This model describes how a positive bond with a credible, caring person creates a sense of accountability, which in turn increases engagement in health behaviors. Text message interaction could enhance, rather than dilute, supportive accountability because individuals tend to make more positive attributions of their communication partners and engage in more self-disclosure when using “lean media” that does not include the voice of the communication partner [13]. These findings, along with current AYA technology practices, suggest CPS could be more acceptable and efficacious if delivered by live, real-time, interactive text messaging with human coaches instead of phone calls.

### Current study

The aim of this randomized pilot trial was to assess the feasibility, acceptability and preliminary efficacy of CPS delivered by phone calls or text messages to AYA with different CHC. We hypothesized that delivering CPS by text message could increase acceptability and medication adherence compared to phone call delivery. This study builds upon our previous pilot work by including a randomized controlled design with a control group. Our study extends the literature on mHealth to promote AYA adherence in several ways. First, although increasingly interactive and personalized automated text message interventions are proliferating in the adherence literature, there have been few randomized trials evaluating the impact of live text message conversations. Second, rather than focusing on a single diagnosis, this study enrolled AYA with different CHC. This approach could produce a more generalizable intervention and greatly increase the number of patients having access to efficacious self-management support, leading to greater reduction in negative healthcare outcomes, costs, and burden. Third, despite comprising over 20% of the US AYA population, Latinx patients make up only 5% of mHealth study populations [14]. The proposed study enrolled a majority of Latinx participants, increasing national representativeness of the broader mHealth literature. Largely due to disparities in social determinants of health [15], Latinx youth in the U.S. are at increased risk for several CHC compared to non-Latinx youth, including cardiometabolic-related conditions and asthma [16]. Therefore, the exclusion of Latinx youth from the mHealth research literature impacts the field’s capacity to develop appropriate and effective illness self-management tools for a population in need of health support.

## Methods

### Setting and sample

**Fig. 1:**
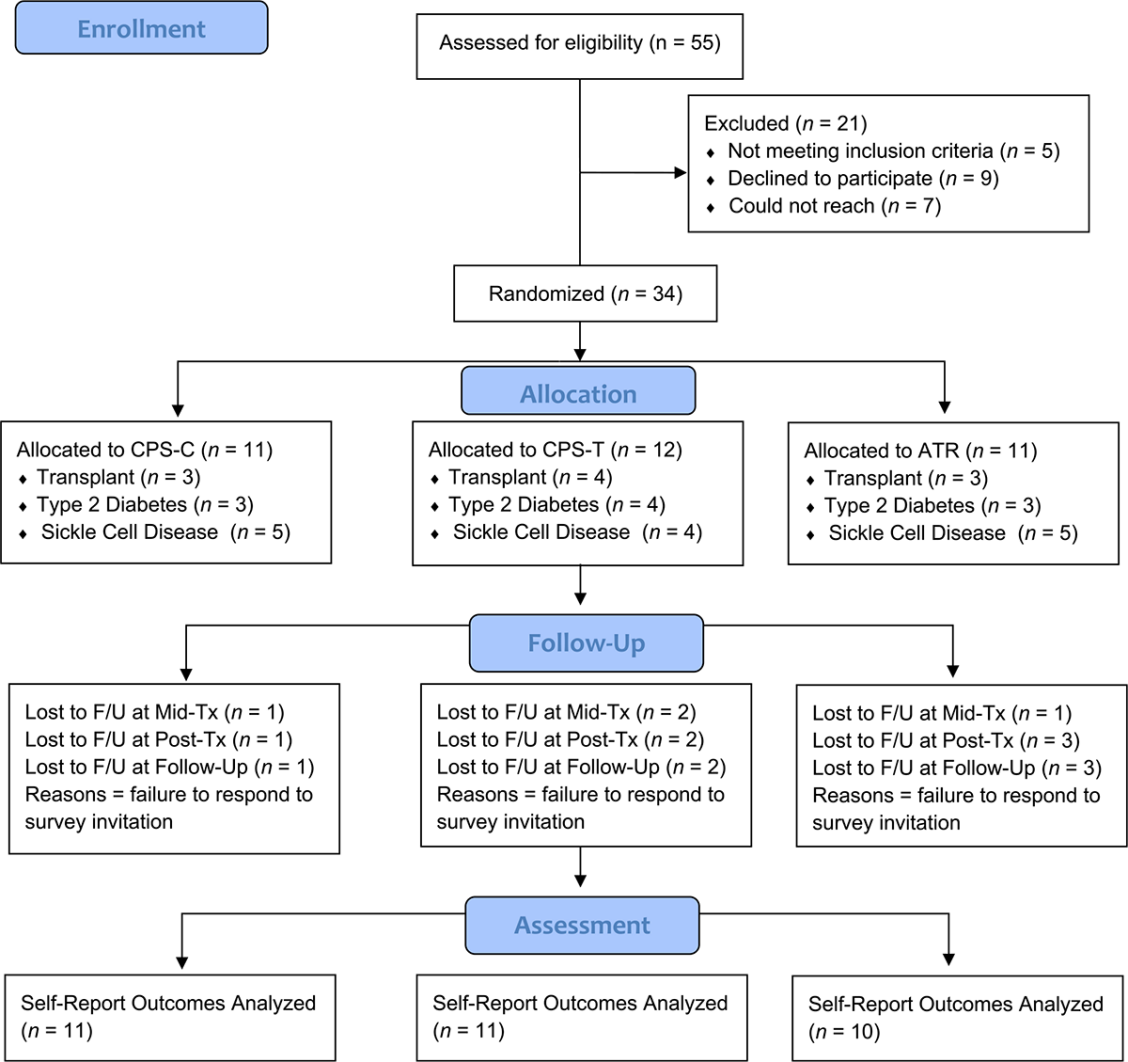
CONSORT diagram

This randomized controlled pilot study (NCT04241627) took place at Children’s Hospital Los Angeles, a pediatric hospital in an urban setting in the United States. Enrollment was open between August 2020 and August 2021. Study enrollment was concluded at this time due to the grant funding timeline. A sample of AYA (*N* = 34) between the ages of 15 and 20 years old with either epilepsy, sickle cell disease, a solid organ transplant, or type 2 diabetes, who were taking at least one oral medication per day, enrolled after direct invitations from their healthcare providers. The study initially aimed to enroll participants with either epilepsy or a solid organ transplant, but due to difficulties meeting enrollment targets, in January 2021, we expanded the inclusion criteria to enroll participants with sickle cell disease and type 2 diabetes, as well. Inclusion criteria were 1) provider and patient agreement that medication adherence is currently < 80%, 2) access to a cell phone, and 3) ability to speak and understand English. The only exclusion criterion was cognitive impairment that precluded participants from engaging in the consent process or study protocol [17]. See CONSORT diagram in Fig. 1.

### Procedures

This study was approved by the Children’s Hospital Los Angeles Institutional Review Board (CHLA-20–00168). Participants were randomly assigned to one of three 12-week interventions in a 1:1:1 ratio: CPS delivered through phone calls (CPS-C), CPS delivered through text messaging (CPS-T), or automated text message reminders (ATR). Automated reminders were selected as the comparison condition based on meta-analytic evidence that this simple, low cost mHealth strategy has small-to-medium-sized effects on adherence [11]. Stratified randomization was conducted in permuted blocks to achieve balance in distribution by gender and diagnosis across trial conditions. A colleague outside of the research team used the RAND function in Excel to create a list of random assignments, with each batch of 12 consecutive participants having four assigned to each of the three conditions in random order. The colleague followed these procedures eight times, crossing four CHC and two gender categories. The colleague then created sealed envelopes with assignment information inside for the principal investigator (PI) to open after each participant completed informed consent, according to their CHC and gender. We planned a priori to use envelopes from female batches if gender non-binary participants enrolled in the study, but all participants indicated either identifying as young men or young women. We did not mask to which condition participants were assigned from participants, providers, or study personnel. All participants were administered questionnaires before randomization (pre-treatment), and at 6 weeks (mid-treatment), 12 weeks (post-treatment), and 18 weeks (follow-up), via REDCap [18]. Participants were compensated with $20 after each study assessment. This study was conducted with guidance from a community advisory board of AYA patients with diverse CHC.

### Interventions

#### Cell phone support

CPS was delivered between 3-to-5 days per week (participants chose the frequency upon enrollment) via phone calls (CPS-C) or synchronous, two-way text messaging (CPS-T) for 12 weeks. Each call or text conversation was focused on coaching participants to adhere to their daily oral medications (e.g., hydroxyurea for sickle cell disease; tacrolimus for solid organ transplant, metformin for type 2 diabetes). Each CPS interaction followed a semi-structured script starting with a check-in about adherence since the last contact, problem-solving support to address barriers, and linkage to referrals if needed. CPS is based on theories of social support, in that the coach provides support for adherence directly to the AYA patient and encourages them to seek support available from their existing networks. Participants could earn monthly incentives worth 40 USD contingent upon answering over 75% of the scheduled calls or text messages. Additional details about the CPS intervention can be found in prior publications [7, 8].

Coaches were undergraduate students enrolled in an experiential research course at University of Southern California. Coaches underwent a background check and completed the CITI Human Subjects Protection course, an online mandated reporter course developed by University of Southern California, and the Praesidium Social Media Safety course. They participated in two 4-hour training sessions to learn the intervention, involving a combination of lectures and role-plays with feedback, followed by 1 hour of group supervision every week throughout the trial. All calls/text message conversations were recorded and forwarded to the PI who completed an 8-item fidelity checklist designed by the CPS developers [7].

#### Automated text reminders

The comparison intervention included automated text message reminders (ATR). If participants were randomized to this intervention, they were asked to select what time(s) of day they wanted to receive text message reminders. Each automated reminder used the same language: “It’s time to take your medicine. Please reply with any text to confirm that you received this message.” Texts were delivered using the automated invitations feature in REDCap [19]. Participants could earn 40 USD monthly incentives contingent upon confirming receipt of over 75% of the messages.

### Measures

#### Demographics and health status

At pre-treatment, participants self-reported their age, gender identity, and racial/ethnic identity. In addition, they completed the Family Affluence Scale II (FAS II), a 4-item scale that can be summed, and used to categorize participants as low, medium, or high socioeconomic status [20].

#### Intervention feasibility and acceptability

Feasibility was evaluated based on the percentage of scheduled contacts in which participants engaged during the 12 weeks of intervention (between 3–7 times per week depending on the intervention). Intervention acceptability was assessed using the ease of use and usefulness subscales of the mHealth app usability questionnaire (MAUQ; [21]). Both subscales have demonstrated high internal consistency and significantly correlate with other established measures of mHealth usability and usefulness [21]. Scores were calculated as an average ranging from 1 to 7, with higher scores indicating greater acceptability.

#### Adherence outcomes

Medication adherence over the past week and past month were measured via self-report visual analogue scales (VAS) at pre-treatment, mid-treatment, post-treatment, and follow-up [22]. The VAS produces scores from 0 to 100% adherent [22]. Referencing the past week and past month, three VAS items were administered, assessing 1) the percentage of the time patients took their medicine (VAS1), 2) the percentage of the time patients took all the doses for the day (VAS2), and 3) the percentage of the time patients took their medicine according to the directions (VAS3). Throughout this article, we refer to VAS scores regarding the past week with a “w” subscript and those regarding the past month with an “m” subscript. Surveys were conducted remotely via REDCap, because administering self-report adherence surveys by computer may decrease social desirability bias [23].

We attempted to confirm the validity of self-report adherence measures with two methods: 1) Medication Event Monitoring System (MEMS) Cap (AARDEX Inc.) adherence assessment, and 2) abstracting variables from the electronic medical record system known to correlate with adherence. Participants and caregivers were given the option to use a MEMS Cap during the study. Micro-electronic circuitry in the MEMS Cap records the dates and times the caps are opened. Participants were oriented to storing one of their medications in this pill bottle during the study and asked to mail back the device at the end of the study. If they mailed back the MEMS Cap, they received 15 USD in compensation. We calculated the percentage of prescribed doses that appeared to have been taken from baseline through mid-treatment, from mid-treatment through post-treatment, and from post-treatment through follow-up. MEMS Caps has shown evidence of validity for assessing adherence with all four patient populations targeted in this study [22–26].

In addition, we abstracted several variables from the electronic medical record over the year prior and post study enrollment. For participants with sickle cell disease, we abstracted mean corpuscular volume (MCV) and fetal hemoglobin (HbF) percentages taken as part of regular care. Higher MCV and HbF percentages are indicators of hydroxyurea adherence and are associated with fewer hospitalizations [24–26]. For participants with transplants, we abstracted all tacrolimus levels from laboratory blood draws collected as part of regular care. We calculated the medication level variability index (MLVI), a validated objective measure of the degree of fluctuation of tacrolimus blood levels over time [27]. MLVI is calculated as the standard deviation of at least 3 tacrolimus trough blood levels for each patient. A higher MLVI denotes more fluctuation in levels and higher values indicate erratic adherence [28]. For participants with type 2 diabetes, we abstracted HbA_1c_ levels. Several studies with adult patients have found a significant relationship between metformin adherence and HbA_1c_ levels [29, 30].

### Analytic plan

We began by calculating descriptive statistics of participant demographics, feasibility and acceptability indicators, self-reported adherence, MEMS Cap data, and EMR variables. To assess the validity of self-reported adherence, we calculated correlation coefficients between VAS scores and MEMS Cap and EMR variables. To take advantage of every available data point and account for repeated measures clustered within individual participants, we ran mixed-effect models in R version 4.2.0, using a nested model-building process. Only participants with at least two data points were included in the analyses (*N* = 32). Primary analyses involved a linear mixed model with fixed study arm and baseline outcome value effects and a random participant effect to account for within-participant correlation. We conducted both models that were unadjusted and then adjusted for covariates. We began by running models evaluating whether assignment to either CPS group (CPS-C or CPS-T) versus ATR predicted better adherence outcomes, and then ran models evaluating whether assignment to CPS-T versus CPS-C predicted better adherence outcomes. We had little missing data and a small sample; therefore, we did not use any weighting methods to address missing data. Given the small sample and pilot nature of this study, we reported the *p*-values associated with tests of significance, but also inspected the results for trends or signals of significance.

### A priori power analysis

Using STATA SE Version 15 software, we estimated we would achieve 87% statistical power achieved with our target sample (*N* = 72), assuming Cohen’s *d* = 0.85 based on our preliminary data [8], specifying within-subject intra-class correlation of 0.5 for repeated measures [29].

## Results

We did not reach our target sample size. However, the primary purpose of pilot studies is to evaluate the feasibility of a novel intervention. Additionally, even if a pilot study is not sufficiently powered to detect statistically significant effects, it can produce estimates of effect sizes to plan for future, larger hypothesis testing studies [31]. Therefore, we proceeded with analyses. Fourteen participants with sickle cell disease, 10 with a transplant, 10 with type 2 diabetes, and zero with epilepsy enrolled in the study. In our previous pilot trial with AYA with transplants, only 22.2% of eligible young men enrolled in the trial, while 87.5% of young women did [8]. In the current study the enrollment rates were much more balanced across gender; 69.2% of eligible young men enrolled and 55.2% of eligible young women enrolled. Participants on average were 17.67 years old (*SD* = 1.39). Of those completing their demographic surveys (*n* = 33), 17 (51.52%) identified as cisgender young men and 16 (48.48%) identified as cisgender young women. See Table 1 for additional participant characteristics.

In both CPS interventions, participants were given the option to choose 3, 4, or 5 contacts a week from their coach. Participants assigned to CPS-C elected to receive 3.73 calls per week on average (*SD* = 0.90). Those in CPS-T chose to receive 3.92 texts on average (*SD* = 0.90). ATR participants received reminders daily, between 1–3 times per day based on the frequency of their medication-taking. Fidelity scores were high in both intervention groups; CPS-C sessions averaged 7.94 (*SD* = 0.25) and CPS-T sessions averaged 7.81 (*SD* = 0.53) on the 8-point scale. No adverse events were detected.

### Intervention feasibility and acceptability

On average over the 12-week intervention, CPS-C participants engaged in 79.04% of their scheduled calls (*SD* = 20.22) and earned 87.27 USD (*SD* = 39.27) in incentives. CPS-T participants engaged in engaged in 78.86% of their scheduled texts (*SD* = 24.45) and earned 96.67 USD (*SD* = 46.58) in incentives. ATR participants confirmed receipt of 59.73% of their scheduled texts (*SD* = 33.48) and earned 47.27 USD (*SD* = 46.71) in incentives. There was no significant difference in the percentage of contacts to which participants responded, *F*(2,31) = 1.95, *p* = 0.16. There was a significant difference in the incentives earned between the three groups, *F*(2,31) = 3.93, *p* = 0.03. A Tukey post hoc test indicated that CPS-T participants earned significantly more incentives than ATR participants, *p* = 0.03. There was no significant difference between CPS-C and ATR participants in incentives earned, *p* = 0.10, or between CPS-C and CPS-T participants, *p* = 0.87.

At mid-treatment, CPS-C participants rated the ease of use of the intervention as *M* = 6.68 (*SD* = 0.37) and usefulness as *M* = 6.54 *(SD* = 0.50), CPS-T participants rated the ease of use as *M* = 6.82 (*SD* = 0.25) and usefulness as *M* = 6.51 (*SD* = 0.68), and ATR participants rated the ease of use as *M* = 6.09 (*SD* = 1.36) and usefulness as *M* = 5.33 (*SD* = 1.50). At post-treatment, CPS-C participants rated the ease of use as *M* = 6.77 (*SD* = 0.36) and usefulness as *M* = 6.65 (*SD* = 0.50), CPS-T participants rated the ease of use as *M* = 6.78 (*SD* = 0.55) and usefulness as *M* = 6.69 (*SD* = 0.45), and ATR participants rated the ease of use as *M* = 6.32 (*SD* = 1.16) and usefulness as *M* = 5.94 (*SD* = 1.62). There was a significant group difference in the mid-treatment usefulness ratings, *F*(2,26) = 4.62, *p* = 0.02. A Tukey post hoc test indicated that participants rated the usefulness of ATR significantly lower than both CPS-C, *p* = 0.04, and CPS-T, *p* = 0.04. There was no statistically significant difference in perceived usefulness between CPS-C and CPS-T at mid-treatment, *p* = 1.00. There were no significant group differences in mid-treatment, *F*(2,25) = 2.00, *p* = 0.16, or post-treatment ease of use ratings, *F*(2,25) = 1.11, *p* = 0.35, or post-treatment usefulness ratings, *F*(2,25) = 1.73, *p* = 0.20.

### MEMS cap and EMR data

Self-reported adherence means and standard deviations for each intervention group at each assessment period are reported in Table 2. There were no significant differences at pre-treatment between the three groups for VAS1_w_, *F*(2,30) = 0.41, *p* = 0.67; VAS2_w_, *F*(2,30) = 0.57, *p* = 0.57; VAS3_w_, *F*(2,30) = 1.17, *p* = 0.33; VAS1_m_, *F*(2,30) = 0.39 *p* = 0.68; VAS2_m_, *F*(2,29) = 0.29, *p* = 0.75; or VAS3_m_, *F*(2,30) = 0.55, *p* = 0.59. VAS scores generally increased over the study period across all three intervention groups.

MEMS Caps scores are also summarized in Table 2. Twenty-two participants (64.71%) agreed to use the MEMS Caps. Only 10 participants returned their MEMS Cap at the end of the study; 2 reported they lost their MEMS Cap and 10 did not respond to multiple reminders by mail and phone to send their MEMS Cap back. We found small non-significant correlations between VAS scores and MEMS Caps scores at mid-treatment (ranging from *r* = 0.06 to *r* = 0.27), post-treatment (ranging from *r* = 0.26 to *r* = 0.28) and follow-up (ranging from *r* = 0.26 to *r* = 0.28). Absolute scores on VAS and MEMS Caps differed substantially (e.g., at mid-treatment, CPS-C participants reported they were 90.25% adherent via the VAS1_m_, but MEMS Caps indicated only 52.00% adherence). MEMS Caps scores indicated decreasing adherence over the course of the study, although when the PI communicated with participants about returning their MEMS Caps by mail, several participants reported they had stopped using the device to store their medications over the course of the study.

Descriptive data from EMR labs related to therapeutic drug monitoring or health status in the year prior and post enrollment in the study are found in Table 3. We expected baseline VAS scores to correlate positively with MCV and HbF% among participants with sickle cell disease, and to correlate negatively with MLVI among participants with transplants and with HbA_1c_ among participants with type 2 diabetes. VAS did correlate positively with HbF% (ranging from *r* = 0.11 to *r* = 0.36) but correlated negatively with MCV (ranging from *r* = −0.21 to *r* = −0.04), although none of these correlations were statistically significant. Counter to expectations, VAS outcomes were positively correlated with MVLI (ranging from *r* = 0.59 to *r* = 0.67). VAS2_w_ (*r* = 0.67, *p* = 0.03), VAS1_m_ (*r* = 0.65, *p* = 0.04), and VAS2_m_ (0.59, *p* < 0.05) were statistically significantly correlated with MLVI, while the remaining were not statistically significant. As expected, VAS outcomes were mostly negatively correlated with HbA_1c_ (ranging from *r* = −0.23 to *r* = 0.07), though all were non-significant. The sample size for EMR data was very small, since variables differed for each diagnosis, precluding statistical analyses of differences in changes over time between intervention groups. Participants with sickle cell disease demonstrated slight increases in MCV and HbF% in all three intervention groups. Participants with transplants demonstrated slight improvement in tacrolimus variability (MLVI) in the CPS-C and ATR groups, but not in the CPS-T group. Participants with type 2 diabetes demonstrated increasing HbA_1c_ in all three groups.

### Intervention effects on self-reported adherence

Table 4 shares the estimates derived from the best-fitting models predicting self-reported adherence examining the effect of assignment to either CPS condition (i.e., CPS-C or CPS-T) versus ATR. Table 5 shares the estimates derived from a similar model examining the effect of assignment to CPS-T versus CPS-C. Change from baseline between-arm differences at mid-treatment, post-treatment, and follow-up are reported as model-based estimates, with corresponding 95% confidence limits and *p*-values. The final models examined the effect of treatment assignment, time as a categorical variable, interaction terms for treatment and time, and covariates (i.e., age, gender, diagnosis). Although age, gender, and diagnosis did not significantly predict outcomes, the linear mixed-models adjusting for these covariates had the best model fit. Therefore, the results from these adjusted models are reported. Models included *n* = 32 participants, excluding two participants who did not complete the VAS items at least twice. The estimates reported in Tables 4 and 5 are those which pertain to the study hypotheses. The full model estimates are available as Supplemental online material.

From baseline to post-treatment, participants in both CPS interventions combined reported a greater increase in adherence compared to participants assigned to ATR. Across the VAS items, CPS participants reported raising their adherence between 8.59 to 25.80 points higher than ATR participants did on the 100-point scale. However, most of these differences are not statistically significant (*p* > 0.05), except for VAS3_w_ (*p* = 0.01). CPS participants did not report consistently better adherence in comparison to ATR participants at mid-treatment or follow-up. Contrary to our hypotheses, from baseline to post-treatment, participants in the CPS-T intervention reported smaller increases in adherence compared to those in the CPS-C intervention. For example, on the VAS1_w_ item, participants in CPS-T reported an increase from baseline to post-treatment of 5.59 points while participants in CPS-C reported an increase of 21.18 points (*p* = 0.14). Most of these differences were not statistically significant, except for VAS3_m_ (*p* = 0.03). From baseline to mid-treatment and baseline to follow-up, CPS-T also reported relatively smaller increases in comparison to CPS-C, with changes on VAS3_w_ significantly different at mid-treatment (*p* = 0.04), and changes on VAS3_m_ significantly different at follow-up (*p* = 0.03).

### Post-hoc power analysis

Due to recruitment difficulties, our sample was less than half the intended size. Only *N* = 32 participants had sufficient data to be included in models. The effect size (differences in change from baseline to post-treatment) between CPS groups and ATR is relatively small (*d* ≈ 0.30) for most VAS items, except for VAS3_w_ (*d* = 0.82). For the smaller difference, our sample size only resulted in statistical power of 22.6%. For the larger effect size (*d* = 0.82) seen in VAS3_w_, the power increased to 71.9%. To achieve power of 80% to detect a small effect size, we would have needed to enroll 164 participants in this study [32]. All calculations were done using the R package “WebPower” and based on a repeated-measures ANOVA (special case of random effects), using a 5% level of significance and moderate correlation between baseline VAS score and follow-up VAS score [33].

## Discussion

In this pilot randomized trial, we observed that self-reported adherence increased in three different types of mHealth intervention, cell phone coaching by phone, cell phone coaching by text, and automated text message reminders. We identified some signals that providing human coaching via phone calls or text messages may promote improved medication adherence among AYA struggling with illness self-management, compared with automated text message reminders. Participants found the cell phone support interventions to be easy to use and potentially more useful than automated reminders. Participants engaged most consistently in cell phone coaching by text message, and slightly less in coaching by phone calls. They were least consistent in confirming receipt of automated text messages. Self-report data was suggestive that cell phone support delivered via phone calls may have improved adherence more than when delivered via text message, despite the popularity of text message communication among AYA. Participants reported particularly strong improvements in taking their medication according to instructions (e.g., on time, with food). These results add to a small but growing literature to support the provision of human coaching to support AYA in the development of self-management skills and preparing for the transition to adult care [34] Considering the lack of strong evidence for adherence-promoting interventions in general [35], this pilot study suggests human support via technology should continue to be considered for inclusion in the clinical toolbox.

Lower than planned recruitment impacted statistical power to detect differences in this study. Further, the small sample size inhibited our ability to assess confirmatory validity by correlating self-reported adherence with MEMS Cap and EMR adherence indicators. Using MEMS Caps in this population proved difficult with many participants either declining to use the method, not adhering to instructions regarding its use, or failing to return the device. Pediatric patients’ difficulty using MEMS Caps has been reported elsewhere [36] suggesting other confirmatory methods may be preferable in this population or participants may need greater levels of instruction or support. The small, but not statistically significant, correlations between MEMS Cap outcomes and self-reported adherence variables provide some support for the validity of the self-report data. However, the disparate absolute values suggest self-reported adherence could be an overestimate, and MEMS Cap data could be an underestimate.

The small numbers of participants with each diagnosis make it difficult to interpret the correlations between self-reported adherence and EMR data. However, generally inspecting the EMR data from the year prior to and post enrollment provides detail helpful for understanding the adherence and health trajectories of participants in this study. Participants with sickle cell disease showed improving MCV and HbF% in all three intervention groups, but their mean values were under the target range (MCV ≥ 100 fl/L; HbF > 20%) [22–24] across the entire study period. It is important to note that environmental factors beyond adherence, such as cold weather, can impact red blood cell labs [37]. Participants with transplants demonstrated slight improvement in tacrolimus variability (MLVI) in the CPS-C and ATR groups, but not in the CPS-T group. However, in both time periods, all groups’ mean MLVI hovered close to the erratic adherence cutoff point (MLVI > 2.0) [28]. Participants with type 2 diabetes demonstrated increasing HbA_1c_ in all three groups, far above the target (HbA_1c_ ≤ 7.0%) [29]. Furthermore, most participants with type 2 diabetes were also prescribed insulin and encouraged to engage in health-promoting behaviors to control their HbA_1c_, while the mHealth interventions in this trial only targeted oral medication taking. Across these diagnostic groups, these EMR variables suggest clinically significant poor adherence before and after the interventions tested in this pilot study. Despite some signals of improving adherence based on self-report data, many participants continued to demonstrate indicators of poor adherence and health status. The lack of meaningful improvement in health status suggests more comprehensive interventions may be necessary, such as multicomponent interventions or behavioral family and individual therapy [38]. However, even if effects are relatively smaller, adding less costly and more scalable mHealth interventions to the broader adherence promotion toolkit could make a meaningful impact of AYA health and wellbeing.

### Limitations and strengths

The results of this study should be considered in light of both its limitations and strengths. Adherence is also notoriously difficult to measure, which limits the certainty of the conclusions we can draw from the self-reported data. However, using several methods to assess adherence (self-report, MEMS caps, electronic health record laboratory values) allows us to consider if results appear consistent across operationalizations. Mixed effects models were more appropriate for this sample size and do benefit from the flexibility to include every datapoint available rather than other models which use listwise deletions. However, due to the small sample, we were not able to test more complex models and it was not appropriate to use weighting methods to address missing data. Stratifying randomization in blocks by gender and focal diagnosis may have helped reduce the effects of confounding. The provision of incentives for engaging in the interventions could have affected how participants rated the usability of the intervention, and poses barriers to these interventions being adopted in real-world clinical settings. Further research is needed to disentangle the role of incentives in mHealth adherence interventions and reduce intervention costs to promote scalability. Finally, this study was a single-site pilot randomized trial conducted during the COVID-19 pandemic, so generalization to other sites or historical periods is limited. However, this study contributes to the mHealth literature by enrolling racially and ethnically diverse AYA which could improve the national representativeness of future systematic reviews and meta-analyses examining mHealth efficacy.

## Conclusions

This pilot trial tested an understudied adherence promotion strategy—human coaching via phone calls and text messages—with the aim of supporting AYA with CHC in developing a key self-management skill. Although, a small sample and challenges in measuring adherence complicate the interpretation of these study results, there are signals that cell phone support may assist some AYA in improving their medication adherence. This study demonstrated that cell phone support was acceptable and feasible across three different medical conditions, indicating it may be broadly applicable to any condition involving oral medication adherence. Further study with larger samples is warranted considering the clinical significance of non-adherence and the lack of sufficient efficacious interventions for promoting self-management during this key developmental period. Future research would benefit from broadening the scope of inquiry to understand how adolescent developmental changes impact engagement in mHealth interventions and adherence behaviors, and how mHealth interventions impact the transition to adulthood.

## Supplementary Material

Suppl Material **Additional file 1: Table S1.** Full Table Predicting VAS1_w_: Both CPS Conditions Combined versus ATR. **Table S2.** Full Table Predicting VAS2_w_: Both CPS Conditions Combined versus ATR. **Table S3.** Full Table Predicting VAS3_w_: Both CPS Conditions Combined versus ATR. **Table S4** Full Table Predicting VAS1_m_: Both CPS Conditions Combined versus ATR. **Table S5.** Full Table Predicting VAS2_m_: Both CPS Conditions Combined versus ATR. **Table S6.** Full Table Predicting VAS3_m_: Both CPS Conditions Combined versus ATR. **Table S7.** Full Table Predicting VAS1_w_: CPS-T versus CPS-C. **Tables S8.** Full Table Predicting VAS2_w_: CPS-T versus CPS-C. **Table S9.** Full Table Predicting VAS3_w_: CPS-T versus CPS-C. **Table S10.** Full Table Predicting VAS1_m_: CPS-T versus CPS-C. **Table S11.** Full Table Predicting VAS2_m_: CPS-T versus CPS-C. **Table S12.** Full Table Predicting VAS3_m_: CPS-T versus CPS-C.

## Figures and Tables

**Table 1:** Baseline participant characteristics

	CPS-C (*n* = 11) *M* (*SD*)	CPS-T (*n* = 12) *M* (*SD*)	ATR (*n* = 11)^a^ *M* (*SD*)
Age	17.82 (1.47)	17.42 (1.62)	17.82 (1.08)
Diagnosis	*n* (%)	*n* (%)	*n* (%)
Sickle cell disease	5 (45.45)	4 (33.33)	5 (50.00)
Transplant	3 (27.27)	4 (33.33)	3 (30.00)
Type 2 diabetes	3 (27.27)	4 (33.33)	3 (30.00)
Gender			
Young men	6 (54.55)	6 (50.00)	5 (50.00)
Young women	5 (45.45)	6 (50.00)	5 (50.00)
Latinx Ethnic Identity	6 (54.55)	8 (66.67)	6 (60.00)
Racial Identity			
White	1 (9.09)	4 (33.33)	0 (0.00)
Black	4 (36.36)	3 (25.00)	2 (20.00)
American Indian/Native	1 (9.09)	0 (0.00)	0 (0.00)
Native Hawaiian/Pacific	1 (9.09)	0 (0.00)	0 (0.00)
Islander			
Asian	0 (0.00)	0 (0.00)	0 (0.00)
More than one race	1 (9.09)	1 (8.33)	4 (40.00)
Unknown	2 (18.18)	0 (0.00)	3 (30.00)
Decline to state	1 (9.09)	4 (33.33)	1 (10.00)
Socioeconomic Status			
Low	2 (18.18)	3 (25.00)	2 (20.00)
Medium	7 (63.64)	5 (41.67)	5 (50.00)
High	2 (18.18)	4 (33.33)	3 (30.00)

a One participant assigned to the ATR condition did not complete any of their surveys. Therefore, percentages are calculated using 10 as the denominator in this column

**Table 2:** Adherence outcomes by intervention and assessment period

	Pre-Tx *M* (*SD*)	Mid-Tx *M* (*SD*)	Post-Tx *M* (*SD*)	Follow-Up *M* (*SD*)
CPS-C	*n* = 11^a^	*n* = 10	*n* = 11	*n* = 10
VAS Previous Week	–	–	–	–
VAS1_w_	70.09 (33.18)	88.10 (19.11)	91.27 (8.70)	94.00 (7.67)
VAS2_w_	78.36 (31.44)	91.20 (13.64)	95.36 (6.53)	93.50 (10.10)
VAS3_w_	59.73 (34.39)	80.00 (24.90)	86.55 (17.58)	91.78 (11.97)
VAS Previous Month	–	–	–	–
VAS1_m_	67.82 (31.66)	90.25 (20.44)	89.91 (14.68)	92.20 (10.47)
VAS2_m_	68.60 (29.76)	88.89 (22.74)	90.91 (15.12)	91.80 (9.90)
VAS3_m_	62.09 (30.62)	82.80 (22.04)	89.82 (16.28)	91.90 (9.93)
MEMS Cap Percentage	–	52.00 (43.50)	40.75 (47.14)	22.75 (39.12)
CPS-T	*n* = 12	*n* = 10	*n* = 10	*n* = 10
VAS Previous Week	–	–	–	–
VAS1_w_	77.67 (29.66)	89.67 (17.46)	84.80 (31.07)	85.00 (30.02)
VAS2_w_	82.50 (30.11)	84.11 (32.69)	86.20 (31.30)	87.60 (30.39)
VAS3_w_	80.00 (30.15)	78.89 (33.98)	86.80 (31.22)	88.80 (29.36)
VAS Previous Month	–	–	–	–
VAS1_m_	72.67 (31.72)	78.50 (31.86)	79.40 (30.04)	83.40 (30.70)
VAS2_m_	75.83 (31.68)	83.20 (22.85)	81.10 (30.27)	85.30 (31.19)
VAS3_m_	75.42 (32.85)	82.50 (20.17)	82.30 (30.49)	85.20 (31.22)
MEMS Cap Percentage	–	32.67 (17.90)	11.67 (12.01)	4.67 (2.52)
ATR	*n* = 10	*n* = 10	*n* = 8	*n* = 8
VAS Previous Week	–	–	–	–
VAS1_w_	66.00 (29.64)	82.80 (25.32)	68.75 (39.03)	82.13 (23.09)
VAS2_w_	68.00 (35.62)	83.40 (21.69)	68.13 (38.96)	81.75 (23.62)
VAS3_w_	72.70 (31.71)	82.11 (19.98)	65.63 (39.46)	81.88 (22.00)
VAS Previous Month	–	–	–	–
VAS1_m_	60.50 (34.18)	81.80 (23.71)	65.88 (37.75)	80.13 (24.10)
VAS2_m_	65.70 (35.47)	81.70 (23.94)	63.13 (36.75)	81.25 (24.42)
VAS3_m_	64.10 (35.45)	81.80 (22.74)	64.63 (35.81)	81.63 (24.39)
MEMS Cap Percentage	–	51.67 (29.02)	14.33 (11.24)	2.67 (2.08)

a The number of participants (*n*) listed in these columns refers to the subgroup completing the VAS items at the time. MEMS Caps were only returned by 10 participants total, from 4 participants who were assigned to CPS-C, 3 to CPS-T, and 3 to ATR

**Table 3:** Electronic medical record variables

	Year Prior to Enrollment	Year Post Enrollment
CPS-C	–	–
Sickle cell disease	*n* = 5	*n* = 4
MCV, *M (SD)*	92.74 (15.64)	96.63 (15.41)
HbF %, *M (SD)*	10.42 (14.62)	12.93 (14.82)
Transplant	*n* = 3	*n* = 3
MLVI, *M (SD)*	1.81 (0.56)	1.73 (0.80)
Type 2 diabetes	*n* = 3	*n* = 3
HbA_1c′_ *M (SD)*	11.77 (2.03)	12.60 (0.24)
CPS-T	–	–
Sickle cell disease	*n* = 4	*n* = 4
MCV, *M (SD)*	93.44 (3.95)	96.31 (5.26)
HbF %, *M (SD)*	5.84 (3.85)	9.02 (6.98)
Transplant	*n* = 4	*n* = 4
MLVI, *M (SD)*	2.49 (1.12)	2.52 (1.85)
Type 2 diabetes	*n* = 4	*n* = 4
HbA_1c′_ *M (SD)*	10.91 (2.08)	12.21 (1.51)
ATR	–	–
Sickle cell disease	*n* = 5	*n* = 3
MCV, *M (SD)*	94.32 (8.21)	96.64 (9.50)
HbF %, *M (SD)*	18.34 (12.43)	20.99 (12.75)
Transplant	*n* = 3	*n* = 3
MLVI, *M (SD)*	2.07 (1.76)	1.75 (1.00)
Type 2 diabetes	*n* = 3	*n* = 3
HbA_1c′_ *M (SD)*	9.93 (0.98)	10.22 (1.04)

**Table 4:** Estimates from best-fitting models predicting self-reported adherence: both CPS conditions combined versus ATR

	Estimates	95% *CI*	*P*	Estimates	95% *CI*	*P*	Estimates	95% *CI*	*P*
Dependent Variable	VAS1_w_			VAS2_w_			VAS3w		
Fixed Effects									
Intercept	26.08	10.34, 41.82	0.002	23.32	6.00, 40.63	0.009	26.59	7.09, 46.09	0.008
CPS * MidTx	−2.08	−21.95, 17.79	0.84	−6.65	−24.19, 10.90	0.46	0.32	−19.53, 20.16	0.98
CPS * PostTx	11.45	−9.14, 32.03	0.28	10.88	−7.36, 29.13	0.24	25.80	5.62, 45.98	0.01
CPS * Followup	−5.21	−25.89, 15.48	0.62	−6.65	−24.98, 11.69	0.48	6.12	−14.26, 26.50	0.56
Observations	118			118			116		
AIC	993.96			981.97			984.22		
Dependent Variable	VAS1_m_			VAS2_m_			VAS3_m_		
Fixed Effects									
Intercept	25.35	6.95, 43.75	0.007	21.31	3.40, 39.23	0.020	26.01	8.63, 43.38	0.994
CPS * MidTx	−9.15	−30.24, 11.95	0.40	−5.49	−24.56, 13.59	0.57	−3.09	−22.12, 15.94	0.75
CPS * PostTx	8.59	−13.21, 30.39	0.44	15.44	−4.34, 35.23	0.13	17.70	−2.17, 37.57	0.08
CPS * Followup	−6.43	−28.33, 15.48	0.57	−5.89	−25.79, 14.00	0.56	−5.33	−25.29, 14.64	0.60
Observations	117			114			119		
AIC	1006.25			955.92			1003.36		

Reference groups are baseline for time and ATR for treatment

**Table 5:** Estimates from best-fitting models predicting self-reported adherence: CPS-T versus CPS-C

	Estimates	95% *CI*	*P*	Estimates	95% *CI*	*P*	Estimates	95% *CI*	*P*
Dependent Variable	VAS1w			VAS2w			VAS3w		
Fixed Effects									
Intercept	31.22	15.90, 46.54	< 0.001	28.94	8.09, 49.79	0.007	18.40	−1.95, 38.75	0.08
CPS * MidTx	−8.68	−30.65, 13.28	0.44	−11.44	−28.23, 5.34	0.18	−21.91	−42.39, −1.42	0.04
CPS * PostTx	−15.60	−36.98, 5.79	0.15	−12.31	−28.57, 3.94	0.14	−18.66	−38.52, 1.20	0.07
CPS * Followup	−16.21	−37.89, 5.47	0.14	−7.22	−23.71, 9.27	0.39	−23.05	−43.54, −2.55	0.03
Observations	82			82			81		
AIC	666.83			646.32			660.83		
Dependent Variable	VAS1m			VAS2m			VAS3m		
Fixed Effects									
Intercept	32.60	13.95, 51.25	0.001	23.75	5.13, 42.37	0.01	27.24	8.45, 46,03	0.994
CPS * MidTx	−17.82	−41.29, 5.65	0.14	−10.71	−29.51, 8.08	0.26	−16.76	−36.16, 2.63	0.09
CPS * PostTx	−17.10	−39.43, 5.23	0.13	−17.93	−36.04, 0.18	0.05	−21.77	−40.88, −2.66	0.03
CPS * Followup	−12.47	−35.14, 10.19	0.28	−11.79	−30.22, 6.64	0.21	−21.05	−40.43, −1.66	0.03
Observations	81			78			83		
AIC	670.35			615.12			671.83		

Reference groups are baseline for time and ATR for treatment

## Data Availability

Available upon request to author, subject to IRB approval.
