# Supplementary material for: Randomized pilot trial of cell phone support to improve medication adherence among adolescents and young adults with chronic health conditions
Source: BMC Digit Health. Author manuscript; Available in PMC 2024 Aug 29. (PMC11360945; doi:10.1186/s44247-024-00069-w)
Supplement: Suppl Material — Additional file 1: Table S1. Full Table Predicting VAS1w: Both CPS Conditions Combined versus ATR. Table S2. Full Table Predicting VAS2w: Both CPS Conditions Combined versus ATR. Table S3. Full Table Predicting VAS3w: Both CPS Conditions Combined versus ATR. Table S4 Full Table Predicting VAS1m: Both CPS Conditions Combined versus ATR. Table S5. Full Table Predicting VAS2m: Both CPS Conditions Combined versus ATR. Table S6. Full Table Predicting VAS3m: Both CPS Conditions Combined versus ATR. Table S7. Full Table Predicting VAS1w: CPS-T versus CPS-C. Tables S8. Full Table Predicting VAS2w: CPS-T versus CPS-C. Table S9. Full Table Predicting VAS3w: CPS-T versus CPS-C. Table S10. Full Table Predicting VAS1m: CPS-T versus CPS-C. Table S11. Full Table Predicting VAS2m: CPS-T versus CPS-C. Table S12. Full Table Predicting VAS3m: CPS-T versus CPS-C. [file NIHMS2018084-supplement-Suppl_Material.docx]

**Online Supplemental Materials**

**File Name:** Online Supplemental Materials

**Title of Data:** Online Supplemental Materials

**Description of Data:** Twelve tables reporting comprehensive statistical output from tests run to analyze the impact of interventions on self-reported medication adherence.

Table S1. Full Table Predicting VAS1_w_: Both CPS Conditions Combined versus ATR

Table S2. Full Table Predicting VAS2_w_: Both CPS Conditions Combined versus ATR

Table S3. Full Table Predicting VAS3_w_: Both CPS Conditions Combined versus ATR

Table S4. Full Table Predicting VAS1_m_: Both CPS Conditions Combined versus ATR

Table S5. Full Table Predicting VAS2_m_: Both CPS Conditions Combined versus ATR

Table S6. Full Table Predicting VAS3_m_: Both CPS Conditions Combined versus ATR

Table S7. Full Table Predicting VAS1_w_: CPS-T versus CPS-C

Tables S8. Full Table Predicting VAS2_w_: CPS-T versus CPS-C

Table S9. Full Table Predicting VAS3_w_: CPS-T versus CPS-C

Table S10. Full Table Predicting VAS1_m_: CPS-T versus CPS-C

Table S11. Full Table Predicting VAS2_m_: CPS-T versus CPS-C

Table S12. Full Table Predicting VAS3_m_: CPS-T versus CPS-C

*Table S1. Full Table Predicting* VAS1_w_*: Both CPS Conditions Combined versus ATR*

|  | Estimates | 95% *CI* | *p* |
| --- | --- | --- | --- |
| Dependent Variable | VAS1_w_ | | |
| Fixed Effects |  |  |  |
| Intercept | 26.08 | 10.34, 41.82 | 0.002 |
| Difference in mean score between intervention and control at baseline | 4.17 | -10.25, 18.59 | 0.572 |
| Difference in mean score between intervention and control at mid-tx | 16.80 | 0.55, 33.05 | 0.043 |
| Difference in mean score between intervention and control at post-tx | 2.30 | -15.03, 19.64 | 0.795 |
| Difference in mean score between intervention and control at follow-up | 17.24 | -0.10, 34.57 | 0.052 |
| Slope of baseline value and outcome for control | 0.60 | 0.46, 0.75 | 0.000 |
| Slope of age and outcome for control | 2.84 | -0.31, 5.99 | 0.077 |
| Difference in mean score between male and female at baseline for control | -2.37 | -10.35, 5.60 | 0.560 |
| Difference in mean score between type 2 diabetes and sickle cell disease at baseline for control | 3.31 | -6.51, 13.13 | 0.509 |
| Difference in mean score between transplant and sickle cell disease at baseline for control | 0.78 | -9.78, 11.34 | 0.886 |
| CPS * MidTx | -2.08 | -21.95, 17.79 | 0.84 |
| CPS * PostTx | 11.45 | -9.14, 32.03 | 0.28 |
| CPS * Followup | -5.21 | -25.89, 15.48 | 0.62 |
| Observations | 118 |  |  |
| AIC | 993.96 |  |  |

*Table S2. Full Table Predicting* VAS2_w_*: Both CPS Conditions Combined versus ATR*

|  | Estimates | 95% *CI* | *p* |
| --- | --- | --- | --- |
| Dependent Variable | VAS1_w_ | | |
| Fixed Effects |  |  |  |
| Intercept | 23.32 | 6.00, 40.63 | 0.009 |
| Difference in mean score between intervention and control at baseline | 4.95 | -9.33, 19.23 | 0.498 |
| Difference in mean score between intervention and control at mid-tx | 15.40 | 1.06, 29,74 | 0.036 |
| Difference in mean score between intervention and control at post-tx | 0.17 | -15.22, 15.55 | 0.984 |
| Difference in mean score between intervention and control at follow-up | 14.30 | -1.09, 29.69 | 0.069 |
| Slope of baseline value and outcome for control | 0.63 | 0.47, 0.78 | 0.000 |
| Slope of age and outcome for control | 1.29 | -2.47, 5.04 | 0.502 |
| Difference in mean score between male and female at baseline for control | 0.87 | -8.40, 10.13 | 0.855 |
| Difference in mean score between type 2 diabetes and sickle cell disease at baseline for control | 4.69 | -7.15, 16.53 | 0.438 |
| Difference in mean score between transplant and sickle cell disease at baseline for control | 1.26 | -10.70, 13.23 | 0.837 |
| CPS * MidTx | -6.65 | -24.19, 10.90 | 0.46 |
| CPS * PostTx | 10.88 | -7.36, 29.13 | 0.24 |
| CPS * Followup | -6.65 | -24.98, 11.69 | 0.48 |
| Observations | 118 |  |  |
| AIC | 981.97 |  |  |

*Table S3. Full Table Predicting* VAS3_w_*: Both CPS Conditions Combined versus ATR*

|  | Estimates | 95% *CI* | *p* |
| --- | --- | --- | --- |
| Dependent Variable | VAS1_w_ | | |
| Fixed Effects |  |  |  |
| Intercept | 26.59 | 7.09, 46.09 | 0.008 |
| Difference in mean score between intervention and control at baseline | -0.72 | -16.28, 14.84 | 0.928 |
| Difference in mean score between intervention and control at mid-tx | 11.68 | -4.70, 28.07 | 0.163 |
| Difference in mean score between intervention and control at post-tx | -8.11 | -25.13, 8.90 | 0.350 |
| Difference in mean score between intervention and control at follow-up | 13.16 | -3.86, 30.18 | 0.130 |
| Slope of baseline value and outcome for control | 0.63 | 0.45, 0.81 | 0.000 |
| Slope of age and outcome for control | 1.34 | -2.82, 5.50 | 0.529 |
| Difference in mean score between male and female at baseline for control | -1.32 | -11.98, 9.34 | 0.809 |
| Difference in mean score between type 2 diabetes and sickle cell disease at baseline for control | 3.57 | -9.72, 16.86 | 0.599 |
| Difference in mean score between transplant and sickle cell disease at baseline for control | -0.78 | -13.74, 12.19 | 0.907 |
| CPS * MidTx | 0.32 | -19.53, 20.16 | 0.98 |
| CPS * PostTx | 25.80 | 5.62, 45.98 | 0.01 |
| CPS * Followup | 6.12 | -14.26, 26.50 | 0.56 |
| Observations | 116 |  |  |
| AIC | 984.22 |  |  |

*Table S4. Full Table Predicting* VAS1_m_*: Both CPS Conditions Combined versus ATR*

|  | Estimates | 95% *CI* | *p* |
| --- | --- | --- | --- |
| Dependent Variable | VAS1_w_ | | |
| Fixed Effects |  |  |  |
| Intercept | 25.35 | 6.95, 43.75 | 0.007 |
| Difference in mean score between intervention and control at baseline | 4.65 | -11.81, 21.10 | 0.580 |
| Difference in mean score between intervention and control at mid-tx | 21.30 | 4.15, 38.45 | 0.015 |
| Difference in mean score between intervention and control at post-tx | 5.36 | -13.01, 23.74 | 0.568 |
| Difference in mean score between intervention and control at follow-up | 20.64 | 2.27, 39.01 | 0.028 |
| Slope of baseline value and outcome for control | 0.58 | 0.39, 0.76 | 0.000 |
| Slope of age and outcome for control | -0.69 | -4.78, 3.39 | 0.740 |
| Difference in mean score between male and female at baseline for control | 1.99 | -8.09, 12.07 | 0.700 |
| Difference in mean score between type 2 diabetes and sickle cell disease at baseline for control | 0.25 | -12,36, 12.86 | 0.970 |
| Difference in mean score between transplant and sickle cell disease at baseline for control | -2.08 | -16.16, 12.00 | 0.773 |
| CPS * MidTx | -9.15 | -30.24, 11.95 | 0.40 |
| CPS * PostTx | 8.59 | -13.21, 30.39 | 0.44 |
| CPS * Followup | -6.43 | -28.33, 15.48 | 0.57 |
| Observations | 117 |  |  |
| AIC | 1006.25 |  |  |

*Table S5. Full Table Predicting* VAS2_m_*: Both CPS Conditions Combined versus ATR*

|  | Estimates | 95% *CI* | *p* |
| --- | --- | --- | --- |
| Dependent Variable | VAS1_w_ | | |
| Fixed Effects |  |  |  |
| Intercept | 21.31 | 3.40, 39.23 | 0.020 |
| Difference in mean score between intervention and control at baseline | 2.97 | -11.65, 17.59 | 0.691 |
| Difference in mean score between intervention and control at mid-tx | 16.00 | 0.54, 31.47 | 0.043 |
| Difference in mean score between intervention and control at post-tx | -3.12 | 19.67, 13.44 | 0.713 |
| Difference in mean score between intervention and control at follow-up | 17.19 | 0.63, 33.74 | 0.042 |
| Slope of baseline value and outcome for control | 0.66 | 0.49, 0.83 | 0.000 |
| Slope of age and outcome for control | 0.17 | -3.41, 3.76 | 0.93 |
| Difference in mean score between male and female at baseline for control | -1.41 | -10.39, 7.58 | 0.759 |
| Difference in mean score between type 2 diabetes and sickle cell disease at baseline for control | 5.30 | -6.49, 17.10 | 0.379 |
| Difference in mean score between transplant and sickle cell disease at baseline for control | 0.12 | -11.56, 11.79 | 0.985 |
| CPS * MidTx | -5.49 | -24.56, 13.59 | 0.57 |
| CPS * PostTx | 15.44 | -4.34, 35.23 | 0.13 |
| CPS * Followup | -5.89 | -25.79, 14.00 | 0.56 |
| Observations | 114 |  |  |
| AIC | 955.92 |  |  |

*Table S6. Full Table Predicting* VAS3_m_*: Both CPS Conditions Combined versus ATR*

|  | Estimates | 95% *CI* | *p* |
| --- | --- | --- | --- |
| Dependent Variable | VAS1_w_ | | |
| Fixed Effects |  |  |  |
| Intercept | 26.01 | 8.63, 43.38 | 0.994 |
| Difference in mean score between intervention and control at baseline | 2.55 | -12.21, 17.30 | 0.736 |
| Difference in mean score between intervention and control at mid-tx | 17.70 | 2.07, 33.33 | 0.03 |
| Difference in mean score between intervention and control at post-tx | -0.48 | -17.23, 16.26 | 0.955 |
| Difference in mean score between intervention and control at follow-up | 22.00 | 5.26, 38.74 | 0.011 |
| Slope of baseline value and outcome for control | 0.60 | 0.44, 0.77 | 0.000 |
| Slope of age and outcome for control | 0.46 | -3.21, 4.12 | 0.81 |
| Difference in mean score between male and female at baseline for control | -1.83 | -11.44, 7.78 | 0.709 |
| Difference in mean score between type 2 diabetes and sickle cell disease at baseline for control | 2.62 | -9.61, 14.84 | 0.676 |
| Difference in mean score between transplant and sickle cell disease at baseline for control | -1.23 | -12.91, 10.44 | 0.836 |
| CPS * MidTx | -3.09 | -22.12, 15.94 | 0.75 |
| CPS * PostTx | 17.70 | -2.17, 37.57 | 0.08 |
| CPS * Followup | -5.33 | -25.29, 14.64 | 0.60 |
| Observations | 119 |  |  |
| AIC | 1003.36 |  |  |

*Table S7. Full Table Predicting* VAS1_w_*: CPS-T versus CPS-C*

|  | Estimates | 95% *CI* | *p* |
| --- | --- | --- | --- |
| Dependent Variable | VAS1_w_ | | |
| Fixed Effects |  |  |  |
| Intercept | 31.22 | 15.90, 46.54 | <0.001 |
| Difference in mean score between intervention and control at baseline | 4.89 | -10.23, 20.01 | 0.526 |
| Difference in mean score between intervention and control at mid-tx | 19.11 | 3.79, 34.43 | 0.015 |
| Difference in mean score between intervention and control at post-tx | 21.18 | 6.26, 36.11 | 0.006 |
| Difference in mean score between intervention and control at follow-up | 20.15 | 4.83, 35.48 | 0.010 |
| Slope of baseline value and outcome for control | 0.50 | 0.30, 0.69 | 0.000 |
| Slope of age and outcome for control | 0.76 | -2.64, 4.15 | 0.663 |
| Difference in mean score between male and female at baseline for control | 3.85 | -6.71, 14.41 | 0.475 |
| Difference in mean score between type 2 diabetes and sickle cell disease at baseline for control | 2.54 | -7.94, 13.02 | 0.635 |
| Difference in mean score between transplant and sickle cell disease at baseline for control | 4.38 | -7.24, 16.00 | 0.46 |
| CPS-T * MidTx | -8.68 | -30.65, 13.28 | 0.44 |
| CPS-T * PostTx | -15.60 | -36.98, 5.79 | 0.15 |
| CPS-T * Followup | -16.21 | -37.89, 5.47 | 0.14 |
| Observations | 82 |  |  |
| AIC | 666.83 |  |  |

*Tables S8. Full Table Predicting* VAS2_w_*: CPS-T versus CPS-C*

|  | Estimates | 95% *CI* | *p* |
| --- | --- | --- | --- |
| Dependent Variable | VAS1_w_ | | |
| Fixed Effects |  |  |  |
| Intercept | 28.94 | 8.09, 49.79 | 0.007 |
| Difference in mean score between intervention and control at baseline | 1.50 | -13.19, 16.19 | 0.841 |
| Difference in mean score between intervention and control at mid-tx | 14.26 | 2.60, 25.91 | 0.017 |
| Difference in mean score between intervention and control at post-tx | 17.00 | 5.69, 28.31 | 0.004 |
| Difference in mean score between intervention and control at follow-up | 11.44 | -0.23, 23.10 | 0.055 |
| Slope of baseline value and outcome for control | 0.58 | 0.32, 0.84 | <0.001 |
| Slope of age and outcome for control | -0.59 | -5.57, 4.39 | 0.816 |
| Difference in mean score between male and female at baseline for control | 4.00 | -10.90, 18.90 | 0.554 |
| Difference in mean score between type 2 diabetes and sickle cell disease at baseline for control | 1.75 | -12.98, 16.48 | 0.817 |
| Difference in mean score between transplant and sickle cell disease at baseline for control | -11.44 | -28.23, 5.34 | 0.182 |
| CPS-T * MidTx | -11.44 | -28.23, 5.34 | 0.18 |
| CPS-T * PostTx | -12.31 | -28.57, 3.94 | 0.14 |
| CPS-T * Followup | -7.22 | -23.71, 9.27 | 0.39 |
| Observations | 82 |  |  |
| AIC | 646.32 |  |  |

*Table S9. Full Table Predicting* VAS3_w_*: CPS-T versus CPS-C*

|  | Estimates | 95% *CI* | *p* |
| --- | --- | --- | --- |
| Dependent Variable | VAS1_w_ | | |
| Fixed Effects |  |  |  |
| Intercept | 18.40 | -1.95, 38.75 | 0.08 |
| Difference in mean score between intervention and control at baseline | 6.75 | -10.74, 24.23 | 0.450 |
| Difference in mean score between intervention and control at mid-tx | 22.79 | 8.55, 37.03 | 0.002 |
| Difference in mean score between intervention and control at post-tx | 26.82 | 13.00, 40.64 | <0.001 |
| Difference in mean score between intervention and control at follow-up | 31.21 | 16.49, 45.94 | <0.001 |
| Slope of baseline value and outcome for control | 0.69 | 0.36, 1.011 | <0.001 |
| Slope of age and outcome for control | 0.84 | -5.29, 6.97 | 0.789 |
| Difference in mean score between male and female at baseline for control | -2.40 | -21.50, 16.70 | 0.806 |
| Difference in mean score between type 2 diabetes and sickle cell disease at baseline for control | 7.37 | -10.25, 24.98 | 0.413 |
| Difference in mean score between transplant and sickle cell disease at baseline for control | -1.75 | -17.17, 13.67 | 0.824 |
| CPS-T * MidTx | -21.91 | -42.39, -1.42 | 0.04 |
| CPS-T * PostTx | -18.66 | -38.52, 1.20 | 0.07 |
| CPS-T * Followup | -23.05 | -43.54, -2.55 | 0.03 |
| Observations | 81 |  |  |
| AIC | 660.83 |  |  |

*Table S10. Full Table Predicting* VAS1_m_*: CPS-T versus CPS-C*

|  | Estimates | 95% *CI* | *p* |
| --- | --- | --- | --- |
| Dependent Variable | VAS1_w_ | | |
| Fixed Effects |  |  |  |
| Intercept | 32.60 | 13.95, 51.25 | 0.001 |
| Difference in mean score between intervention and control at baseline | 3.05 | -13.93, 20.04 | 0.725 |
| Difference in mean score between intervention and control at mid-tx | 21.91 | 4.75, 39.08 | 0.013 |
| Difference in mean score between intervention and control at post-tx | 22.09 | 6.53, 37.64 | 0.006 |
| Difference in mean score between intervention and control at follow-up | 20.51 | 4.49, 36.54 | 0.013 |
| Slope of baseline value and outcome for control | 0.45 | 0.19, 0.71 | 0.001 |
| Slope of age and outcome for control | -3.94 | -8.29, 0.42 | 0.077 |
| Difference in mean score between male and female at baseline for control | 9.76 | -2.78, 22.29 | 0.128 |
| Difference in mean score between type 2 diabetes and sickle cell disease at baseline for control | -1.61 | -15.50, 12.28 | 0.821 |
| Difference in mean score between transplant and sickle cell disease at baseline for control | 2.26 | -14.76, 19.28 | 0.795 |
| CPS-T * MidTx | -17.82 | -41.29, 5.65 | 0.14 |
| CPS-T * PostTx | -17.10 | -39.43, 5.23 | 0.13 |
| CPS-T * Followup | -12.47 | -35.14, 10.19 | 0.28 |
| Observations | 81 |  |  |
| AIC | 670.35 |  |  |

*Table S11. Full Table Predicting* VAS2_m_*: CPS-T versus CPS-C*

|  | Estimates | 95% *CI* | *p* |
| --- | --- | --- | --- |
| Dependent Variable | VAS1_w_ | | |
| Fixed Effects |  |  |  |
| Intercept | 23.75 | 5.13, 42.37 | 0.01 |
| Difference in mean score between intervention and control at baseline | 2.93 | -11.43, 17.28 | 0.690 |
| Difference in mean score between intervention and control at mid-tx | 16.28 | 2.44, 30.13 | 0.022 |
| Difference in mean score between intervention and control at post-tx | 21.40 | 8.48, 34.32 | 0.002 |
| Difference in mean score between intervention and control at follow-up | 17.45 | 4.08, 30.82 | 0.011 |
| Slope of baseline value and outcome for control | 0.63 | 0.39, 0.88 | <0.001 |
| Slope of age and outcome for control | -2.08 | -6.41, 2.27 | 0.349 |
| Difference in mean score between male and female at baseline for control | 3.03 | -9.68, 15.75 | 0.641 |
| Difference in mean score between type 2 diabetes and sickle cell disease at baseline for control | 0.98 | -11.60, 13.56 | 0.88 |
| Difference in mean score between transplant and sickle cell disease at baseline for control | 0.51 | -12.59, 13.62 | 0.940 |
| CPS-T * MidTx | -10.71 | -29.51, 8.08 | 0.26 |
| CPS-T * PostTx | -17.93 | -36.04, 0.18 | 0.05 |
| CPS-T * Followup | -11.79 | -30.22, 6.64 | 0.21 |
| Observations | 78 |  |  |
| AIC | 615.12 |  |  |

*Table S12. Full Table Predicting* VAS3_m_*: CPS-T versus CPS-C*

|  | Estimates | 95% *CI* | *p* |
| --- | --- | --- | --- |
| Dependent Variable | VAS1_w_ | | |
| Fixed Effects |  |  |  |
| Intercept | 27.24 | 8.45, 46,03 | 0.994 |
| Difference in mean score between intervention and control at baseline | 6.94 | -9.09, 22.97 | 0.397 |
| Difference in mean score between intervention and control at mid-tx | 22.92 | 9.22, 36.63 | 0.002 |
| Difference in mean score between intervention and control at post-tx | 27.73 | 14.42, 41.03 | <0.001 |
| Difference in mean score between intervention and control at follow-up | 27.28 | 13.58, 40.99 | <0.001 |
| Slope of baseline value and outcome for control | 0.54 | 0.27, 0.81 | <0.001 |
| Slope of age and outcome for control | -1.40 | -6.45, 3.64 | 0.586 |
| Difference in mean score between male and female at baseline for control | 3.46 | -12.03, 18.95 | 0.662 |
| Difference in mean score between type 2 diabetes and sickle cell disease at baseline for control | -0.12 | -15.72, 15.51 | 0.990 |
| Difference in mean score between transplant and sickle cell disease at baseline for control | -1.84 | -16.11, 12.43 | 0.801 |
| CPS-T * MidTx | -16.76 | -36.16, 2.63 | 0.09 |
| CPS-T * PostTx | -21.77 | -40.88, -2.66 | 0.03 |
| CPS-T * Followup | -21.05 | -40.43, -1.66 | 0.03 |
| Observations | 83 |  |  |
| AIC | 671.83 |  |  |
